# Early Side Effects of rhTSH-Aided Radioiodine Ablation and Treatment of Differentiated Thyroid Cancer: A Real-World Experience

**DOI:** 10.1055/s-0046-1823122

**Published:** 2026-06-04

**Authors:** Ismaheel O. Lawal, Priya D. John, Margaret Lim, Ashley Bridges, Gbenga O. Popoola, Saima Muzahir

**Affiliations:** 1 Department of Radiology and Imaging Sciences, Emory University School of Medicine, Atlanta, Georgia, United States; 2 Division of Endocrinology, Metabolism and Lipids, Department of Medicine, Emory University School of Medicine, Atlanta, Georgia, United States; 3 Department of Radiology and Imaging Sciences, Emory University School of Medicine, Atlanta, Georgia, United States; 4 Saxon Court Lincolnshire Partnership NHS Foundation Trust (LPFT), Lincoln, Lincolnshire, United Kingdom

**Keywords:** radioactive iodine, differentiated thyroid cancer, rhTSH, nausea, salivary gland damage, lacrimal gland damage

## Abstract

**Purpose:**

We aimed to investigate the early side effects of recombinant human thyroid-stimulating hormone (rhTSH)-aided radioactive iodine (RAI) administration in an unselected cohort of patients with differentiated thyroid cancer (DTC) in the real-world setting.

**Methods:**

During follow-up (at 6 weeks, 6 months, and 12 months post-RAI) of patients with DTC who received rhTSH-aided RAI, we used a standardized questionnaire to collect patient-reported side effects of RAI. We investigated the factors that impact the occurrence of RAI-associated side effects.

**Results:**

A total of 111 patients with a female predominance (66.7%) and a mean age of 51.1 ± 16.0 years were included, including 13 patients (11.7%) who had previously received RAI. The predominant tumor histology was papillary thyroid cancer, with 91.0% of patients having stage I/II disease. At 6-week follow-up, loss of taste/smell, nausea, salivary gland pain or swelling, dry mouth, and neck pain were reported by 45.9, 37.8, 30.6, 36.9, and 20.7% of patients, respectively. In most patients, these side effects lasted for less than 1 week. At 6-month post-RAI, 13 patients reported lingering RAI-associated side effects, including 4 and 3 patients who reported dry mouth and dry eyes, respectively. A 12-month post-RAI, one patient each reported dry eyes, watery eyes, and fatigue as RAI side effects. Women were at higher risk of neck pain, while young patients were more likely to experience nausea ( *p* < 0.05).

**Conclusion:**

rhTSH-aided RAI administration in DTC patients is associated with tolerable side effects, most of which resolved within weeks of treatment.

## Introduction

Radioactive iodine (RAI) administration is recommended after total thyroidectomy for intermediate- to high-risk differentiated thyroid cancer (DTC) for remnant thyroid tissue ablation, adjuvant treatment of micrometastases, and treatment of known metastatic disease. 1 Radioactive iodine treatment (RAIT) reduces the risk of disease recurrence and thyroid cancer mortality in patients with intermediate- to high-risk DTC. 2 3 RAIT has been used in DTC treatment for many decades, and the side effects of treatment are well known. The early side effects of RAIT are mostly related to the uptake of RAI by the sodium-iodine symporter, including the lacrimal glands, salivary glands, and gastric mucosa. Therefore, side effects of RAIT in the acute and subacute settings include changes in the salivary and lacrimal glandular function, neck pain due to radiation effect in the remnant thyroid tissue, and gastric lining irritation. 4 Elevated serum levels of thyroid-stimulating hormone (TSH) are necessary to optimize thyroid and thyroid cancer uptake of RAI, especially given the relatively low iodine-trapping capability of thyroid cancer cells. 5 Traditionally, elevated serum TSH has been achieved with 4 to 6 weeks of thyroid hormone withdrawal (THW). The resultant severe hypothyroid symptoms from THW can be quite debilitating and not tolerable for some patients. 6

In December 2007, the U.S. Food and Drug Administration approved recombinant human TSH (rhTSH, Thyrogen) as an adjunctive treatment for RAI ablation in patients with DTC after total or near-total thyroidectomy. rhTSH given in two intramuscular injections is now routinely used as an alternative to THW to achieve elevated TSH before RAI administration, eliminating the severe morbidity associated with hypothyroidism and its negative impact on quality of life. 7 Outcomes of postthyroidectomy RAI ablation are similar between elevated TSH achieved by THW versus rhTSH injections. 7 8 Also, rhTSH is associated with lower whole-body radiation dose due to improved RAI biokinetics without compromising uptake in the remnant thyroid tissue. 9 The resultant reduced whole-body RAI residence time has the theoretical benefit of reduced radiation dose to other organs with iodine-trapping capability. Despite these theoretical benefits, the early side effects of rhTSH-aided RAI ablation and treatment of patients with DTC have not been comprehensively investigated in routine clinical settings. A randomized clinical trial comparing the success of ablation between 30 and 100 mCi of RAI found no significant difference in the side effects of THW versus rhTSH-aided RAI ablation. 10 However, another randomized trial reported a higher incidence of some hypothyroid symptoms at the time of RAI administration in patients who received RAI ablation with THW compared with rhTSH. 11 These data may have limited application in a real-world setting given the restricted activity of RAI administered and the highly selected patient population recruited into clinical trials. A few studies that have reported the side effects of rhTSH-aided RAI ablation and treatment in DTC have either focused on salivary and lacrimal glandular toxicities alone or investigated only the long-term complications of RAI administration. 12 13 Therefore, a clinical need exists to investigate the early side effects of RAI administration in the real-world setting of the current rhTSH era. This study aimed to investigate the early side effects of RAI administered with rhTSH stimulation for remnant ablation, adjuvant treatment, and metastatic disease treatment in patients with DTC in a real-world setting.

## Methods

This is a retrospective analysis of prospectively collected side-effect data in patients with DTC treated with rhTSH-aided RAI at our institution. The Institutional Review Board of our institution approved the study and waived the need for formal patient consent due to the retrospective nature of the work. We reviewed the medical records of all patients aged 18 years and older who were treated with RAI for DTC. Inclusion criteria included histological diagnosis of thyroid cancer, status post total/near-total thyroidectomy, and administration of RAI after two doses of 0.9 mg Thyrogen given intramuscularly 24 hours apart. Patients who had been previously treated with RAI were also included. We excluded patients who received RAI with THW for TSH stimulation.

### RAI Administration for Ablation and Treatment

Patient preparation and treatment with RAI-131 were done according to published guidelines. 1 4 Briefly, all patients were evaluated during a clinical consultation to determine their suitability for RAIT. If RAI was deemed suitable, information on the nature of the treatment, alternatives to RAI in DTC treatment, possible side effects, radiation safety precautions, and the treatment goal was provided to patients. Subsequently, consent to proceed with RAIT was obtained after addressing the patient's concerns. All patients observed a 2-week low-iodine diet before undergoing RAIT. A 0.9 mg Thyrogen was administered on days 1 and 2. A diagnostic dose of oral iodine-123 was administered on day 2 using a standard activity of 1 to 2 mCi (37–74 MBq). Whole-body imaging with/without single-photon positron emission imaging/computed tomography imaging was performed on day 3 to determine the extent of remnant thyroid tissue, stage the disease, and guide empirical iodine-131 dosing. Briefly, for empirical RAI dosing, we administer 100 mCi (3700 MBq) to patients with organ-confined disease without regional nodal or distant metastatic disease. Doses of 150 mCi (5,550 MBq) and 200 mCi (7,400 MBq) are administered to patients with positive neck nodes and distant metastatic disease, respectively.

Serum qualitative beta-human chorionic gonadotropin was assayed to rule out pregnancy in all patients of childbearing potential. Iodine-131 was administered orally, while solid food was withheld for at least 1 hour thereafter. Patients were encouraged to drink plenty of liquids orally. Antiemesis with ondansetron 4 mg PO was prescribed for use approximately 1 hour before RAI administration and as needed subsequently. Patients were encouraged to suck lemon candies starting 24 hours after RAIT to increase the saliva flow rate and reduce RAI salivary gland residence time. 14 A low-iodine diet was recommended for an additional 3 days after iodine-131 administration.

### Patient Follow-up and Determination of RAIT-Induced Early Side Effects

In October 2020, we introduced a follow-up plan for all patients who received RAI for DTC within the Emory Healthcare system, where every patient was evaluated with a structured questionnaire (Supplementary File) for side effects of RAI administration at 6 weeks, 6 months, and 12 months after treatment in a nuclear medicine physician-led teleconsult. The questionnaire consists of 22 questions to determine the early side effects of RAIT, such as nausea and neck pain, salivary gland toxicity, lacrimal gland damage, and a general section asking for any health changes occurring after RAIT. Also, questions relating to the observed side effects, preventive measures, such as using ondansetron for nausea/vomiting prevention and the use of sour candy, were included. Individual patient responses to the questions were documented in their electronic medical record. At 6 and 12 months after RAI administration, there was a follow-up teleconsult by a nuclear medicine physician to inquire about the resolution or persistence of their RAI-induced side effects and the occurrence of new side effects. For the purpose of this study, we considered acute and subacute side effects of RAI occurring from treatment administration up to 1 year after RAI as early side effects of RAI.

### Data Analysis

We performed descriptive statistical analysis of all clinicopathologic characteristics of the patients. We used frequency and percentage to report categorical variables. Mean (standard deviation, SD) and median (interquartile range, IQR) were reported for numeric variables as appropriate. We determined the prevalence of the different side effects of RAI administration and the patient adherence to preventive measures using frequency and percentage. We determined the impact of prior RAI administration, the administered RAI activity, age, and gender on the incidence of RAI-associated side effects using the chi-square test or Fisher's exact test, as appropriate. We also used chi-square test or Fisher's exact test to determine if significant differences exist in the incidence of RAI-associated side effects among patients who observed the side-effect-mitigating measures compared with those who did not. We set statistical significance at a *p* -value of <0.05 and performed statistical analysis with IBM SPSS Statistics version 27.

## Results

Between October 2020 and November 2023, 118 patients received RAIT for DTC at our institution. We excluded 7 patients who received RAIT with THW, leaving a total of 111 patients analyzed in this study. The majority of the patients were women (66.7%), with a mean age of 51.1 ± 16.0 years for the entire study population. The predominant histological tumor type was papillary (80.2%), and the disease was mostly stages I/II (91%). Thirteen of 111 patients (11.7%) had been previously treated with RAI (median activity = 104.0 mCi [IQR:30.3–160.0]), including one patient who previously received RAI twice. The median cumulative dose of RAI for the entire study population was 152.0 (IQR: 129.6–176.8) mCi. Table 1 shows the summary baseline clinicopathologic characteristics of the patients.

**Table 1:** Demographic and clinical characteristics

Variable	Frequency	Percentage
Age (y)		
≤ 45	40	36.0
> 45	71	64.0
Mean ± SD	51.13 ± 16.00	
Range	24–90	
Gender		
Women	74	66.7
Men	37	33.3
Histological thyroid cancer subtype		
Follicular carcinoma	4	3.6
Hurthle cell carcinoma	11	9.9
Papillary carcinoma	89	80.2
Papillary carcinoma + poorly differentiated carcinoma	1	0.9
Papillary carcinoma, follicular carcinoma	1	0.9
Poorly differentiated carcinoma	5	4.5
Stage		
I	64	57.7
II	37	33.3
III	3	2.7
IV	1	0.9
IVA	1	0.9
IVB	2	1.8
NA	3	2.7
Dose of RAI in index treatment (mCi)		
< 150	43	38.7
≥ 150	68	61.3
Median (IQR)	151.70 (128.4–175.0)	
Previous RAI dose (mCi)		
Median (IQR)	104.0 (30.3–160.0)	
Cumulative dose (mCi)		
Median (IQR)	152.0 (129.6–176.8)	

Abbreviations: NA, not available; RAI, radioactive iodine; SD, standard deviation.

### Side Effects of Radioactive Iodine Treatment at 6-Week Follow-up

Twenty-three of 107 patients reported neck pain after RAI treatment. In the majority of affected patients, neck pain started within 24 hours of RAI administration and lasted for less than 1 week. Out of 107 patients, 42 patients experienced nausea, lasting for less than 1 week in the majority of them. Among 74 patients who responded to the question on nausea prophylaxis, 57 affirmed the use of ondansetron for nausea prevention. Most patients (82 of 85 patients) reported regular use of sour candy or lemon drops for salivary gland protection, starting 24 hours after RAI treatment, and most continued to use it for more than 24 hours. However, 34 of 105 patients experienced salivary gland pain or swelling, mostly affecting the parotid gland alone ( *n* = 12) or both parotid and submandibular glands ( *n* = 9), with an onset within and after 24 hours in 12 and 19 patients, respectively. At 6 weeks after receiving RAI, 41 patients (36.9%) reported dry mouth as a side effect of treatment, and 7 patients needed to drink water or other fluids while eating. Loss of taste or smell was the most common patient-reported side effect of RAI, reported by 51 patients (45.9%), with an onset within the first week of treatment lasting more than 2 weeks in the majority of the affected patients. Eye symptoms were rare, with 12 patients (10.8%) reporting eye redness or increased tearing within the first 6 weeks of treatment. Most patients adhered to the oral hydration recommendation ( Table 2 ). Other symptoms reported by the patients are shown in Supplementary Table S1 . Notable other symptoms reported by the patients were fatigue in 11 patients and easy bruising or bleeding (nosebleed, gum bleeding, and menorrhagia) in 7 patients.

**Table 2:** Incidence of side effects of radioactive iodine administration and the preventive measures observed

Variable	Frequency	Percentage
Neck pain		
Yes	23	20.7
No	84	75.7
NR	4	3.6
Time of onset ( *n* = 23)		
Within 24 h after treatment	13	56.5
More than 24 h after treatment	6	26.1
NR	4	17.4
Pain duration ( *n* = 23)		
Less than a week	15	65.2
More than a week	5	21.7
NR	3	13.0
Nausea		
Yes	42	37.8
No	65	58.6
NR	4	3.6
Nausea duration ( *n* = 42)		
Less than a week	34	30.6
More than a week	7	6.3
NR	1	0.9
Medication for nausea		
Yes	58	52.3
No	17	15.3
NR	36	32.4
Salivary gland pain/swelling		
Yes	34	30.6
No	71	64.0
NR	6	5.4
Salivary gland regions involved ( *n* = 34)		
Parotid	12	35.3
Parotid and submandibular	9	26.5
Submandibular	9	26.5
NR	4	11.8
Time of onset of salivary gland pain/swelling ( *n* = 34)		
Within 24 h after treatment	12	35.3
More than 24 h after treatment	19	55.9
NR	3	8.8
Duration of salivary gland pain/swelling ( *n* = 34)		
A day or less	5	14.7
Less than a week	19	55.9
A week or more	6	17.6
Ongoing	2	5.9
Dry mouth		
Yes	41	36.9
No	65	58.6
NR	5	4.5
Able to eat with drinking water/fluids		
Yes	87	78.4
No	7	6.3
NR	17	15.3
Use of sour/lemon candy		
Yes	82	73.9
No	3	2.7
NR	26	23.4
Duration of use of candy ( *N* = 84)		
Less than a week	38	46.3
A week or more	34	41.5
As Instructed'	3	3.7
NR	7	8.5
Loss of taste/smell		
Taste	35	31.5
Taste and smell	2	1.8
Yes (not specified)	14	12.6
No	55	49.5
NR	5	4.5
Time of onset of loss of taste/smell ( *n* = 51)		
Between 1 and 2 wk after treatment	13	25.5
Within 1 wk after treatment	31	60.8
NR	7	13.7
Duration of loss of taste/smell		
0–2 wk	20	39.2
3 wk or more	27	52.9
NR	4	7.8
Red eyes/tearing		
Yes	12	10.8
No	93	83.8
NR	6	5.4
Quantity oral hydration		
8 or fewer glasses/day	23	20.7
More than 8 glasses/day	84	75.7
NR	4	3.6
Duration of hydration ( *n* = 107)		
Less than a week	27	25.2
1 wk or more	76	71.0
NR	4	3.7

Abbreviation: NR, no response.

### Side Effects of Radioactive Iodine Treatment at 6- and 12-Month Follow-ups

At the 6-month follow-up, 13 patients reported at least one lingering or new side effect of RAI, including 2 patients with more than one symptom. The common symptoms were dry mouth and dry eyes in four and three patients, respectively ( Table 3 ).

**Table 3:** Side effects of radioactive iodine treatment at 6-month follow-up

Side effects of RAI	Frequency
Dry mouth	4
Dry eyes	3
Altered/loss of taste	1
Easy bruising	1
Parotid gland discomfort with chewing	1
Worsening hot flushes	1
Persistent cough	1
Mouth sores	1
Tonsillolith	1
Bluish discoloration in the back of the throat	1
On and off tightness behind the left ear	1

At 12-month follow-up, only three patients had symptoms they felt were secondary to RAI treatment, including dry eyes ( *n* = 1), watery eyes ( *n* = 1), and fatigue ( *n* = 1).

### Factors Influencing the Incidence of Side Effects of Radioactive Iodine Administration

We investigated the influence of gender on the incidence of the different side effects of RAI administration and found a higher incidence of salivary gland pain or swelling in women relative to men (43.7 vs. 8.8%, *p* < 0.001). The incidence of loss of taste or smell was similarly higher among women than men (57.7 vs. 28.6%, *p* = 0.003) ( Table 4 ). The incidence of other side effects of RAI administration, including neck pain, nausea, dry mouth, and red eyes/excessive tearing, was not significantly different between women and men ( Table 4 ).

**Table 4:** Impact of gender on the incidence of side effects of radioactive iodine administration

Variable	Gender			*χ* ^2^	*p* -Value
	Female	Male	Total		
	*n* (%)	*n* (%)	*n* (%)		
Salivary gland pain/swelling					
Yes	31 (43.7)	3 (8.8)	34 (32.4)	12.744	**<0.001**
No	40 (56.3)	31 (91.2)	71 (67.6)		
Neck pain					
Yes	17 (23.9)	6 (16.7)	23 (21.5)	0.750	0.387
No	54 (76.1)	30 (83.3)	84 (78.5)		
Nausea					
Yes	32 (45.1)	10 (27.8)	42 (39.3)	2.996	0.083
No	39 (54.9)	26 (72.2)	65 (60.7)		
Dry mouth					
Yes	27 (38.0)	14 (40.0)	41 (38.7)	0.038	0.845
No	44 (62.0)	21 (60.0)	65 (61.3)		
Loss of taste/smell					
Taste	25 (35.2)	10 (28.6)	35 (33.0)	12.870 ^F^	**0.003**
Taste and smell	2 (2.8)	0 (0.0)	2 (1.9)		
Yes (not specified)	14 (19.7)	0 (0.0)	14 (13.2)		
No	30 (42.3)	25 (71.4)	55 (51.9)		
Red eyes/tearing					
Yes	9 (12.9)	3 (8.6)	12 (11.4)	0.423 ^F^	0.747
No	61 (87.1)	32 (91.4)	93 (88.6)		

Note: *χ* ^2^ , chi-square test; F, Fisher's exact test. Values shown in bold indicate statistical significance ( *p* -Value < 0.05).

In our investigation of the impact of age on the incidence of side effects of RAI administration, nausea was more prevalent among patients aged 45 years and younger than patients older than 45 years (53.8 vs. 30.9%, *p* = 0.019) ( Table 5 ). The incidence of the other side effects of RAI administration was not significantly different between younger (≤45 years) and older (> 45 years) patients ( Table 5 ).

**Table 5:** Impact of age on the incidence of side effects of radioactive iodine administration

Variable	Age (y)			*χ* ^2^	*p* -Value
	≤ 45	> 45	Total		
	*n* (%)	*n* (%)	*n* (%)		
Salivary gland pain/swelling					
Yes	13 (33.3)	21 (31.8)	34 (32.4)	0.026	0.873
No	26 (66.7)	45 (68.2)	71 (67.6)		
Neck pain					
Yes	10 (25.6)	13 (19.1)	23 (21.5)	0.625	0.429
No	29 (74.4)	55 (80.9)	84 (78.5)		
Nausea					
Yes	21 (53.8)	21 (30.9)	42 (39.3)	5.481	**0.019**
No	18 (46.2)	47 (69.1)	65 (60.7)		
Dry mouth					
Yes	17 (44.7)	24 (35.3)	41 (38.7)	0.916	0.338
No	21 (55.3)	44 (64.7)	65 (61.3)		
Loss of taste/smell					
Taste	11 (29.7)	24 (34.8)	35 (33.0)	1.386 ^F^	0.708
Taste and smell	0 (0.0)	2 (2.9)	2 (1.9)		
Yes (not specified)	6 (16.2)	8 (11.6)	14 (13.2)		
No	20 (54.1)	35 (50.7)	55 (51.9)		
Red eyes/tearing					
Yes	2 (5.4)	10 (14.7)	12 (11.4)	2.048 ^F^	0.207
No	35 (94.6)	58 (85.3)	93 (88.6)		

Note: *χ* ^2^ , chi-square test; F, Fisher's exact test.

There was a paradoxical observation of an increased incidence of nausea among patients who used ondansetron for nausea prophylaxis compared with those who did not, with nausea reported by 63.8% of patients who used ondansetron for nausea prophylaxis compared with 17.6% of patients who did not take ondansetron ( *p* = 0.001).

We found no significant difference in the incidences of the different side effects of RAI administration between patients receiving their first-ever RAI and those previously treated with RAI ( *p* > 0.05) ( Supplementary Table S2 ). Similarly, the incidence of the different RAI-associated side effects was not significantly different between patients treated with <150 mCi and those who received ≥150 mCi of RAI in the index ablation or treatment of DTC ( Supplementary Table S3 ). The incidence of salivary gland pain/swelling or subsequent development of dry mouth was not significantly impacted by the use of sour candy or lemon drops administered for salivary gland protection ( Supplementary Table S4 ).

## Discussion

Using prospectively collected data in a real-world setting, we investigated the early side effects of RAI administration for DTC management in the rhTSH era. At 6 weeks post-RAI administration, loss of taste or smell was the most common RAI-associated side effect, reported by 45.9% of all patients, and typically starting within the first week of RAI administration in the majority of the affected patients. Other notable RAI-associated side effects within 6 weeks of administration were nausea, salivary gland pain or swelling, dry mouth, and neck pain, which were reported by 37.8, 30.6, 36.9, and 20.7%, respectively. The incidence of eye changes, including redness and tearing, was relatively rare, seen in only 10.8% of patients.

Elevated serum TSH levels increase RAI uptake by thyroid cancer cells, improving the outcome of RAI administration in DTC management. 15 16 17 The use of rhTSH to achieve elevated serum TSH has become the standard of care in DTC management with RAI. In addition to eliminating the serious morbidities associated with severe hypothyroidism due to THW for serum TSH elevation, rhTSH improves the kinetics of RAI in vivo by reducing the residence time of RAI in other organs, such as salivary glands and lacrimal glands, thereby reducing the absorbed dose to these organs. 9 The incidence of side effects of RAI administration between patients who received RAI aided by rhTSH injection compared with THW has been investigated in some phase 3 trials. However, patients recruited into clinical trials are often super-selected and are not representative of the patients treated in real-world settings. 10 11 A few retrospective studies have also investigated the side effects of RAI in the rhTSH era. 13 18 These retrospective studies are limited in that the occurrence of these RAI-related side effects was not actively elicited from the patients, which could have led to significant underreporting. Our study, therefore, provides a unique contribution to the literature by reporting the incidence of RAI-associated side effects in DTC patients who received RAI aided by rhTSH, using data prospectively collected in a routine care setting. Our study recruited a diverse patient population with respect to age, gender, disease stage, histological subtype, and prior receipt of RAI, reflecting the typical patient population referred for RAI administration for DTC management in the routine clinical care setting.

RAI is generally safe, and most of the clinical side effects it causes are tolerable and transient. In our study, at 6 months following RAI administration, persisting side effects were generally rare. Some notable side effects at this time point were dry mouth, dry eyes, altered taste, and parotid gland discomfort with mastication, seen in 3.6, 2.7, 0.9, and 0.9%, respectively. In the retrospective study by Simões-Pereira et al, sialadenitis occurred in 7.4% of patients who received RAI for the treatment of metastatic DTC. 19 However, the timing of the assessment of side effects was not specified. In our study, we used a physician-administered questionnaire to document RAI-associated side effects. In a retrospective study that utilized salivary gland ultrasound to evaluate for long-term (more than 12 months) RAI-induced salivary gland damage, structural damage of the salivary gland was found in 18.6% of patients who had rhTSH-aided RAI therapy versus 27.2% of patients who had THW-aided RAI therapy ( *p* = 0.037). 20 While this study indicates the greater sensitivity of ultrasound for detecting RAI-induced salivary gland damage, the clinical implications of the ultrasound-detected structural changes remain to be determined.

Patients are generally advised to take certain prophylactic measures to improve the safety profile of RAI administration. One such measure is the prophylactic use of antiemetics to reduce the incidence of nausea and vomiting. In this series, 52.3% affirmed to taking ondansetron for emesis prophylaxis. We found a significantly higher rate of nausea among patients who took antinausea medication compared with those who did not (63.8 vs. 17.6%, *p* = 0.001). In prescribing antiemetics for patients receiving RAI, we generally advise the patients to use the medication if they think they may experience nausea. Therefore, patients who generally experience nausea after taking oral medications were more likely to have used the prescribed ondansetron, while patients who generally have a lower risk of nausea from oral medication did not take the antiemetic medication and still had a lower incidence of nausea after RAI administration. Loss of taste/smell was the most common side effect in this series, reported by 45.9% of the study population. Most of the patients included in this study were treated during the COVID-19 pandemic. We speculate that subclinical infection with the COVID-19 virus, known to present with loss of taste/smell, could have contributed to a higher incidence of this symptom in our series.

Our study had many strengths, including actively asking about the common side effects of RAI administration using a standardized questionnaire in a physician-led teleconsult. We evaluated the patients at three time points, starting at 6 weeks post-RAI administration. This schedule of patient evaluation allows us to capture the early symptoms due to RAI administration, which has hitherto been poorly investigated, especially in the rhTSH era. Moreover, evaluating patients at multiple time points allowed for the temporal evolution of the acute side effects of RAI, which were documented at the 6-week follow-up. However, our study also has some limitations. For example, we evaluated the patients for their clinical symptoms post-RAI administration. Certain known side effects of RAI, such as hematologic toxicity, are better evaluated with laboratory tests. Seven patients (6.3%) reported increased bleeding/easy bruising at the 6-week follow-up. We cannot ascribe these side effects to platelet count or function, as we did not routinely obtain complete blood counts for patient follow-up. Another limitation of our study is the lack of a head-to-head comparison of the incidence and the temporal evolution of these early RAIT side effects among patients treated under rhTSH versus THW. Unfortunately, the majority of patients treated with RAI at our institution do so aided by rhTSH, with only a few patients treated aided by THW. Therefore, we do not have enough patients who received THW-aided RAI for a robust comparison. We did not use a standardized scale, such as the Common Terminology Criteria for Adverse Events, to quantify the severity of treatment-related side effects experienced by patients. However, our qualitative approach to severity quantification showed that the symptoms were mild, tolerable, and transient.

## Conclusion

The early side effects of rhTSH-aided RAI administration for DTC management in the routine clinical care setting include nausea, salivary gland pain or swelling, dry mouth, neck pain, and loss of taste/smell, lasting 1 week or less. Patient-reported side effects of RAI administration are rare at 6 months post-RAI administration and beyond. Women are more likely to experience salivary gland pain or swelling, while younger patients are more likely to experience nausea after RAI administration.
