# Supplementary material for: Early Side Effects of rhTSH-Aided Radioiodine Ablation and Treatment of Differentiated Thyroid Cancer: A Real-World Experience
Source: World J Nucl Med. 2026 Jun 4;25(2):128–35. doi: 10.1055/s-0046-1823122 (PMC13327741; doi:10.1055/s-0046-1823122)
Supplement: Supplementary file 1 — Supplementary Material [file 10-1055-s-0046-1823122-s25100013.pdf]

Supplementary File: Study Questionnaire

CLINICAL INDICATION: thyroid cancer status post total thyroidectomy and iodine-131 ablation therapy.

Date of radioactive iodine treatment with (Thyrogen) or (Hormone withdrawal):

COVID-19 status:

1. Did you feel any neck pain after radioactive iodine treatment? Yes/No
2. If yes, then when did the pain start?
   a. Within 1 day after treatment (within 24 hours of treatment)
   b. Later (more than 24 hours of treatment)
3. How long did the pain last?
   a. Less than a week
   b. More than a week (more than 1 week but less than 2 weeks)
   c. Other (more than 2 weeks)
4. Did you experience nausea after the radioactive iodine treatment? Yes/No
5. If yes, then how long did the nausea last?
   a. Less than a week
   b. More than a week (more than 1 week but less than 2 weeks)
   c. Other (more than 2 weeks)
6. Did you take antinausea medication?
7. If yes, what antinausea medication?
8. Did you suffer from pain or swelling in the salivary glands (parotid/submandibular) after radioactive iodine treatment? Yes/No
9. Which regions were involved?
   a. Parotid (in front of both ears)
   b. Submandibular (under the jaw)
   c. Both parotid and submandibular
10. When did the pain/swelling start?
    a. Within 1 day after treatment (within 24 hours of treatment)
    b. Later (more than 24 hours of treatment)
11. How long did the pain or swelling last?
    a. Less than a week
    b. More than a week (more than 1 week but less than 2 weeks)
    c. Other (more than 2 weeks)
12. Did you use sour candies on a regular basis following treatment? Yes/No
13. If yes, how long did you use them?
    a. Less than 24 hours
    b. 24 hours
    c. 48 hours
    d. 72 hours
14. Did your mouth become dry after radioactive iodine treatment? Yes/No
15. Were you able to eat without having to drink water or any other fluid to help with swallowing? Yes/No
16. Did you lose sense of taste or smell after radioactive iodine therapy? Yes/No
17. When did you lose your sense of taste or smell?
    a. Within 2 to 3 days after treatment
    b. Later (after 4 days after treatment)
18. How long did the loss of sense of smell or taste last?
    a. Less than a week
    b. More than a week (more than 1 week but less than 2 weeks)
    c. Other (more than 2 weeks)
19. Did you suffer often or continuously from red eyes or increased tearing? (Did you experience continuous reddening of the eye or increased tearing after radioactive iodine treatment?) Yes/No
20. How much water did you drink after radioiodine therapy?
    a. More than 8 glasses/day
    b. 6 to 7 glasses/day
    c. 4 to 5 glasses/day
    d. 3 to 4 glasses/day
21. How long did you maintain good hydration following therapy?
    a. 7 days
    b. 5 to 6 days
    c. 3 to 4 days
    d. 1 to 2 days
22. Any other complaints?

Overall experience:

 © 2026. The Author(s).

Supplementary Table S1 Other symptoms reported by the patients at 6-week follow-up

None (has acid peptic disease, takes omeprazole 40 mg/day. Avoiding caffeine and other acidic foods has helped improve his symptoms)
Developed symptoms related to COVID-19 5 days after RAI and tested positive 4 d later
Mild occasional dull ache near the right hip
Mild to moderate diarrhea for 3–4 days after the treatment
Bleeding through nose whenever blowing nose, lasted almost a week. Patchy black discoloration on the tongue 3–4 days after treatment without pain, swelling, or tenderness in tongue that resolved within a week
Tightness and pain on the right side of the neck. Sensation of not being able to breathe
Fatigue for 2 days after treatment
Nosebleeds that began 1 week after treatment, on/off for 3 weeks
Decreased energy and appetite shortly after therapy, which has since resolved
Worsening constipation
Mild bleeding from gums
Sinus tachycardia within a few days of treatment
Easy bruising
Hard to sing. Excessive salivation. Feels like food is going down slowly
Blisters in the nose and lips—day 6 after RAI. Applied Neosporin and Vaseline, have resolved
Mouth sores, took Voltrax & Abreva. (Patient has a history of developing mouth sores.)
Sensitivity of the tongue to spicy and sweet flavors
Left-sided neck pain, saliva feels more abundant, drinking water worsens nausea, and hoarseness. Patient feels these are more related to preexisting conditions
Fatigue, increased hair loss, lost 50 lbs because food was not pleasurable due to metallic taste
Lump in throat, present after thyroidectomy, slightly increased after RAI. Occasional headaches over the past month
Developed COVID-19 shortly after RAI
Mild fatigue that started 1 week after therapy and has persisted.
Low energy
Fatigue on the day after therapy
Fatigue. Diarrhea for 6–7 weeks
Fatigue
Nasal dryness and bleeding for at least 2 weeks. Resolved with nasal saline and Petraglia products
Fingernail and gum discoloration. A few nosebleeds a few weeks ago
Still nauseous. Pain in the T7 vertebra
A headache that occurred 2 days after therapy has resolved. There is also a rash on one arm, recent vision strain, and some posterior cheek discomfort
Chronic hoarseness not related to RAI
Headaches but has had these before therapy
Some difficulty sleeping, but it has not significantly changed with RAI therapy
Hoarse. Heavy periods. Fatigue, which has been improving
Decreased salivation
Fatigue, tongue feels rough
Nerve pain during the weekend after therapy. Some dry mouth in the morning
Occasional fatigue (may be due to raising a toddler)
Continued altered taste, submandibular puffiness
Body aches starting on day 2 for 3–4 days<br>Increased heartburn that improved with Prilosec may be related to other factors
Raspy voice, occasional tenderness on the left side of the neck. Occasional fatigue
Discomfort in the right submandibular gland region related to sialolithiasis

 © 2026. The Author(s).

Supplementary Table S2 Impact of prior radioactive iodine administration on the incidence of RAI-associated side effects

Variable	Previous RAI administration			χ^2	*p*-Value
	Yes	No	Total		
	n (%)	*n* (%)	*n* (%)		
Salivary gland pain/swelling					
Yes	3 (25.0)	31 (33.3)	34 (32.4)	0.337^{F}	0.747
No	9 (75.0)	62 (66.7)	71 (67.6)		
Neck pain					
Yes	1 (7.7)	22 (23.4)	23 (21.5)	1.671^{F}	0.291
No	12 (92.3)	72 (76.6)	84 (78.5)		
Nausea					
Yes	4 (30.8)	38 (40.4)	42 (39.3)	0.447	0.504
No	9 (69.2)	56 (59.6)	65 (60.7)		
Dry mouth					
Yes	4 (30.8)	37 (39.8)	41 (38.7)	0.391	0.532
No	9 (69.2)	56 (60.2)	65 (61.3)		
Loss of taste/smell					
Taste	4 (30.8)	31 (33.3)	35 (33.0)	2.677^{F}	0.478
Taste and smell	0 (0.0)	2 (2.2)	2 (1.9)		
Yes (not specified)	0 (0.0)	14 (15.1)	14 (13.2)		
No	9 (69.2)	46 (49.5)	55 (51.9)		
Red eyes/tearing					
Yes	3 (23.1)	9 (9.8)	12 (11.4)	1.989^{F}	0.168
No	10 (76.9)	83 (90.2)	93 (88.6)		

Abbreviation: RAI, radioactive iodine.
Note: χ^2, chi-square test; F, Fisher's exact test.

 © 2026. The Author(s).

Supplementary Table S3 Impact of administered activity of radioactive iodine on the incidence of RAI-associated side effects

Variable	Dose of RAI in index treatment			χ^2	*p*-Value
	< 150 mCi	≥ 150 mCi	Total		
	n (%)	*n* (%)	*n* (%)		
Salivary gland pain/swelling					
Yes	14 (33.3)	20 (31.7)	34 (32.4)	0.029	0.865
No	28 (66.7)	43 (68.3)	71 (67.6)		
Neck pain					
Yes	11 (25.6)	12 (18.8)	23 (21.5)	0.711	0.399
No	32 (74.4)	52 (81.2)	84 (78.5)		
Nausea					
Yes	16 (37.2)	26 (40.6)	42 (39.3)	0.126	0.723
No	27 (62.8)	38 (59.4)	65 (60.7)		
Dry mouth					
Yes	15 (34.9)	26 (41.3)	41 (38.7)	0.439^{F}	0.507
No	28 (65.1)	37 (58.7)	65 (61.3)		
Loss of taste/smell					
Taste	15 (35.7)	20 (31.2)	35 (33.0)	2.509^{F}	0.498
Taste and smell	1 (2.4)	1 (1.6)	2 (1.9)		
Yes (not specified)	3 (7.1)	11 (17.2)	14 (13.2)		
No	23 (54.8)	32 (50.0)	55 (51.9)		
Red eyes/tearing					
Yes	5 (11.9)	7 (11.1)	12 (11.4)	0.016^{F}	1.000
No	37 (88.1)	56 (88.9)	93 (88.6)		

Abbreviation: RAI, radioactive iodine.
Note: χ^2, chi-square test; F, Fisher's exact test.

Supplementary Table S4 Impact of the use of sour candy or lemon drops on the incidence of salivary gland side effects

Variable	Use of sour candy/lemon drops			χ^2	*p*-Value
	Yes	No	Total		
	n (%)	*n* (%)	*n* (%)		
Salivary gland pain/swelling					
Yes	27 (33.3)	1 (33.3)	28 (33.3)	0.000^{F}	1.000
No	54 (66.7)	2 (66.7)	56 (66.7)		
Dry mouth					
Yes	35 (43.2)	1 (33.3)	36 (42.9)	0.115^{F}	1.000
No	46 (56.8)	2 (66.7)	48 (57.1)		
Eat without drinking water/fluid					
Yes	63 (91.3)	3 (100.0)	66 (91.7)	0.285^{F}	1.000
No	6 (8.7)	0 (0.0)	6 (8.3)		

Note: χ^2, chi-square test; F, Fisher's exact test.

 © 2026. The Author(s).
